# Development of an Indicator Film Based on Cassava Starch–Chitosan Incorporated with Red Dragon Fruit Peel Anthocyanin Extract

**DOI:** 10.3390/polym14194142

**Published:** 2022-10-03

**Authors:** Rianita Pramitasari, Levina Natasha Gunawicahya, Daru Seto Bagus Anugrah

**Affiliations:** 1 Food Technology Study Program, Faculty of Biotechnology, Atma Jaya Catholic University of Indonesia, Tangerang 15345, Indonesia; 2 Biotechnology Study Program, Faculty of Biotechnology, Atma Jaya Catholic University of Indonesia, Tangerang 15345, Indonesia

**Keywords:** film indicator, red dragon fruit peel anthocyanin, cassava starch, chitosan, shrimp freshness monitoring

## Abstract

The increase in new technology and consumer demand for healthy and safe food has led to the development of smart packaging to help consumers understand food conditions in real time. The incorporation of red dragon fruit peel anthocyanin into cassava starch and chitosan films was used in this study as a color indicator to monitor food conditions. This indicator film was generated using the solvent-casting method. The mechanical, morphological, and physicochemical characterizations of the film were studied, and food freshness monitoring was carried out. The results showed that adding red dragon fruit peel anthocyanin increased up to 94.44% of the antioxidant activity. It also improved its flexibility, indicated by the lowest tensile strength (3.89 ± 0.15 MPa) and Young’s modulus (0.14 ± 0.01 MPa) and the highest elongation at break (27.62 ± 0.57%). The indicator film was sensitive to pH, which was indicated by its color change from red to yellow as pH increased. The color of the film also changed when it was used to test the freshness of packaged shrimp at both room and chiller temperatures. According to the results, the indicator film based on cassava starch–chitosan incorporated with red dragon fruit peel anthocyanin showed its potential as a smart packaging material.

## 1. Introduction

Currently, the development of food packaging is related to biotechnology and nanotechnology, which can produce smart packaging with indicators, sensors, and data carriers. In addition, the increased consumer demand for healthy and safe food also encourages the development of smart packaging to help consumers understand the conditions and quality of food in real time [1]. The ability of smart packaging to monitor the food condition supports the trend in sustainability that concerns reducing food waste and loss in the food supply chain [1,2]. Smart packaging is a type of packaging that can monitor the conditions of packaged food and environmental conditions in the packaging, such as pH, temperature, humidity, and spoilage compounds, to provide information about the quality of the food in the package without having to open the package [3,4]. Of the several types of smart packaging, indicator films are one of the simplest types and are usually equipped with a natural or synthetic dye indicator incorporated on a polymer-based film that is responsive to pH and temperature [4,5,6,7].

The use of natural colorants such as anthocyanins has been widely developed as a film color indicator [8,9,10]. Anthocyanins are natural pigments of a class of flavonoid compounds that are sensitive to changes in pH [11,12]. At pH 1–7, anthocyanins are in flavylium cation, carbinol pseudobase, and quinonoidal base forms. With increasing pH, they are transformed to anionic quinonoidal base and chalcone forms. The transformation of chemical structures will change their color [13]. The freshness of foodstuffs, such as meat and shrimp, can be detected due to enzymatic reactions producing volatile base compounds that can interact with anthocyanins and change the color of the indicator [14,15]. Red dragon fruit peel anthocyanin is interesting to be developed as an indicator because its use can reduce the amount of organic waste. Moreover, it is sensitive to pH and can be used in antioxidant and antibacterial applications [16].

Anthocyanin incorporated into starch-based films has been developed to monitor food freshness [17]. Although the use of starch provides high economic value because it is inexpensive and easy to obtain, starch-based films have low elasticity and heat stability and are easily soluble in water [18]. The addition of chitosan and plasticizer in the form of glycerol is able to form a composite film that is more elastic, strong, heat-stable, and not easily penetrated by water [19,20]. In addition, chitosan has antioxidant and antibacterial properties [21], and is able to control the release of bioactive compounds, making its use in indicator films able to increase the responsiveness to pH [22]. Although the synergy between the materials used to make the film is predicted to produce a good film, the use of red dragon fruit peel anthocyanin in the film as an indicator must still be researched.

This research aimed to develop and characterize an indicator film based on cassava starch–chitosan incorporated with red dragon fruit peel anthocyanin extract since the blending of these materials has not been studied to monitor food conditions. The success of film development will be understood based on its ability to be applied to measure the freshness of shrimp as a food product that is susceptible to damage by microorganisms and can produce volatile base compounds that can increase the pH in the headspace of the packaging.

## 2. Materials and Methods

### 2.1. Materials

Red dragon fruit (*Hylocereus polyrhizus*), white leg shrimp (*Litopenaeus vannamei*), and tapioca starch (Rose Brand) were purchased from AEON Mall BSD City, Tangerang, Indonesia. Low-molecular-weight chitosan (≤10 kDa) with a degree of deacetylation of 80–85% was purchased from Xi’an Rongsheng Biotechnology, Xi’an, China. Ethanol, potassium chloride, hydrochloride acid, acetic acid glacial, and 2.2-diphenyl-1-picrylhydrazyl (DPPH) were purchased from Smart-Lab, Tangerang, Indonesia. Glycerol and ammonia were purchased from Merck KGaA, Darmstadt, Germany. Nutrient agar and nutrient broth were purchased from Himedia, Mumbai, India. Other materials were bacterial culture *Escherichia coli* (ATCC^®^ 25922^TM^), *Staphylococcus aureus* (ATCC^®^ 25923^TM^), and distilled water. All chemicals used in this research were analytical grade.

### 2.2. Methods

#### 2.2.1. Extraction of Anthocyanin from the Red Dragon Fruit Peel

Red dragon fruit was washed and its peel was separated from the flesh. The peel of the red dragon fruit was mashed using a food processor (Philips 2061, Shanghai, China) until mushy. After that, as much as 100 g of the peel was weighed. Then, it was put into a solution of 70% ethanol of as much as 500 mL and stirred homogeneously. The mixture was macerated for 24 h in a chiller (5 °C). After maceration, the macerated extract was filtered using cheesecloth. The filtrate obtained was then concentrated using a rotary evaporator (R-300 Buchi, Flawil, Switzerland) at a temperature of 55 °C, a speed of 60 rpm, and a pressure of 130 mbar [9].

#### 2.2.2. Analysis of Total Anthocyanin Content of the Extract

The extract was mixed into buffer solutions of both pH 1 and 4.5. The absorbance of the mixture was measured using a UV-Vis spectrophotometer (Thermo Fisher Scientific, Waltham, MA, USA). The total anthocyanins content was carried out using the following formula [23]:

$$C=\frac{A\times MW\times DF\times 1000}{\varepsilon \times L}$$

where *A* = (*A_520_* − *A_700_*) pH 1 − (*A_520_* − *A_700_*) pH 4.5 and *A* is absorbance, *MW* = the molecular weight of anthocyanins which is 449.2 g/mol, *DF* = the dilution factor, ε = the molar attenuation coefficient of anthocyanins, which is 26,900 L/mol.cm, and *L* = the width of the cuvette in cm.

#### 2.2.3. Analysis of the Color Change of the Extract in Various pH Conditions

A total of 1 mL of the anthocyanin extract was mixed with 1 mL of pH 1–13 buffer each. The result of the color change was then photographed [24].

#### 2.2.4. Preparation of the Films

The formulations of the four types of composite films were prepared, as shown in Table 1.

The making of four composite films began with the preparation of chitosan and cassava starch solution. A 1% (*w/v*) chitosan solution was prepared by mixing 1 g of chitosan powder into 200 mL of acetic acid 1% using a hotplate stirrer (Heidolph MR Hei-Standard, Germany) at 500 r/mins stirring for 24 h. A 2% (*w/v*) cassava solution was prepared by mixing 2 g of cassava starch powder into 200 mL of distilled water. The mixture was then gelatinized using a hotplate stirrer (Thermo Fisher Scientific, Shanghai, China) until it reached a temperature of 80 °C while stirring using a magnetic stirrer.

In making SChA and SCh films, the chitosan solution was mixed with cassava starch solution. After mixing, the solution was stirred using a homogenizer (Heidolph, Schwabach, Germany) at 530 rpm for 10 min. Ch and S films were made according to the formulation without first being homogenized. The finished solution was added to glycerol and homogenized using a homogenizer (T10 ultra-turrax, Selangor, Malaysia) at a speed of 14,500 rpm for 5 min. After that, the anthocyanin extract was added to the SChA indicator film solution and stirred using a stirring bar [25,26,27].

#### 2.2.5. Composite Film Casting

The four variations of the film solution were poured into 6 cm (diameter) plastic Petri dishes with as much as 20 mL in each. Then, the solution was dried in a fume hood for 48 h. After the solution had dried, the film was removed from the plastic Petri dish using a spatula and stored in a closed dark container at 5 °C [19].

#### 2.2.6. Measurement of Thickness and Mechanical Properties

Analysis was performed using a texturometer (Agrosta, Normandy, France). The film thickness was measured using a micrometer with an accuracy of 0.01 mm to determine its thickness. After that, a 4 cm × 1.5 cm film was attached to the pull lever. A tensile test was carried out using an initial grip separation of 1 cm and a crosshead speed of 0.8 mm/s. The analysis was performed at room temperature (25 °C). Tensile strength, elongation at break, and Young’s modulus as measurements of film strength, film flexibility, and film stiffness, respectively, were determined using the formula [16]:

$$Tensile\;strength\;\left(MPa\right)=\frac{F}{A_{0}}\;$$

$$Elongation\;at\;break\;\left(\%\right)=\frac{\delta \;}{L_{0}}\times 100$$

$$Modulus\;young\;\left(MPa\right)=\frac{Tensile\;strength}{Elongation\;at\;break}$$

where *F* = the force applied to the film, *A*_0_ = the area of the film, δ = the increase in length, and *L*_0_ = the initial length of the film.

#### 2.2.7. Composite Films’ Surface Color Analysis

The surface color of the four films was analyzed using a colorimeter (NH310, Shenzhen 3NH Technology Co., LTD., Shenzhen, China). After testing, the values of L, a, and b of the composite films were obtained. The total color difference (∆*E*) was calculated using the following equation [24]:

$$∆E=\sqrt{{\left(L_{s}-L\right)}^{2}+{\left(a_{s}-a\right)}^{2}+{\left(b_{s}-b\right)}^{2}}$$

where *L* = lightness, *a* = red (+) and green (−), *b* = yellow (+) and blue (−), ∆*E* = *L*, *a*, *b* values obtained compared to the standard color (white) of the colorimeter.

#### 2.2.8. Sensitivity Analysis of Indicator Film to Ammonia Vapor

The SChA film was cut into 1 cm × 1 cm sections. After that, the sample was affixed approximately 1 cm from a dark vial tube containing 20 mL of 15 mM ammonia. The indicator film was then left for 7 h at 25 °C. The color change of the indicator film was observed every 1 h. The values of R, G, and B were measured using the Paint program on Windows. Lastly, the sensitivity of the indicator film to ammonia was calculated using the following formula [24]:

$$S_{RGB}\;\left(\%\right)=\frac{\left(R_{i}-R_{f}\right)+\left(G_{i}-G_{f}\right)+\left(B_{i}-B_{f}\right)\;}{R_{i}+G_{i}+B_{i}}\times 100$$

where *R* = red, *G* = green, and *B* = blue. *R_i_*, *G_i_*, and *B_i_* = the initials of *R*, *G*, and *B* values. *R_f_*, *G_f_*, and *B_f_* = the *R*, *G*, and *B* values of the indicator film after the test was conducted.

#### 2.2.9. Sensitivity Analysis of the Indicator Film to Various pH Conditions

The SchA film was cut into 1.5 cm × 1 cm sections. Then, the film was immersed in a buffer solution with a pH of 1–13 for 2 min. The result of the color change was then photographed [24].

#### 2.2.10. Morphology Analysis of Composite Films

The composite films were cut into 2 cm × 2 cm sections and coated with gold. The surface morphology of the composite films was observed using scanning electron microscopy (SEM) (Hitachi SU3500, Tokyo, Japan) with an electric voltage of 20 kV and high vacuum conditions (10^−2^–10^−3^ Pa) [16].

#### 2.2.11. Transparency Analysis

The film was cut into a 3 cm × 0.3 cm section and pasted on the clear part of the cuvette. The light of transmittance was read at a wavelength of 600 nm using a UV-Vis spectrophotometer (Shimadzu UV-2450, Kyoto, Japan). The film transparency was calculated using the formula [24]:

$$Transparency=\frac{logT600}{D}\;$$

where *T*_600_ = the percentage of light transmittance at a wavelength of 600 nm, and *D* = the thickness of the film. The transmittance of the film samples was measured using a UV-Vis spectrophotometer (Shimadzu UV-2450, Kyoto, Japan) at a wavelength of 200–800 nm [24].

#### 2.2.12. Analysis of Antioxidant Activity

A total of 3.9 mL of DPPH solution with a concentration of 0.003 mM was mixed with 0.1 mL of film solution (25 mg of film sample/5 mL of distilled water). The solution mixture was then homogenized using a vortex (Thermofischer, Shanghai, China) and incubated for 30 min in a dark room at 25 °C. The absorbance was then measured at a wavelength of 517 nm using a UV-Vis spectrophotometer (Thermo Fisher Scientific, Waltham, MA, USA). Antioxidant activity was calculated using the formula [24]:

$$Antioxidant\;activity\;\left(\%\right)=\frac{A_{DPPH}-A_{film}\;}{A_{DPPH}}\times 100$$

where *A_DPPH_* = the absorbance of DPPH control in the form of a solution of DPPH and distilled water, and *A_film_* = the DPPH absorbance of the film.

#### 2.2.13. Analysis of Antimicrobial Activity

Analysis of the antimicrobial activity of the film was carried out by dripping 50 L of film solution aseptically on a sterile disc placed in a petri dish containing nutrient agar (NA) which had been inoculated with 0.2 mL of 10^6^ CFU/mL of *Escherichia coli* and *Staphylococcus aureus* in each. After that, the Petri dishes containing the film were incubated at 37 °C for 24 h. The resulting clear zone around the film indicated the presence of bacterial inhibition or antimicrobial properties of the film [25].

#### 2.2.14. Shrimp Freshness Monitoring

A total of 10 g of shrimp was put in a transparent plastic container. The SChA indicator film (1.5 cm × 1 cm) was affixed under the lid of the plastic container, and the film could not be in direct contact with the shrimp. After that, the prepared samples were stored at various temperature treatments, namely room temperature (25 °C) for 5 h and chiller temperature (5 °C) for five days. At room temperature treatment, color changes were observed and photographed every hour. In the chiller treatment, color changes were observed and photographed everyday [26].

#### 2.2.15. Statistical Analysis

All experiments in making the films were repeated three times. Each of these experiments was also characterized and monitored for shrimp freshness three times. The data obtained from the analysis were processed statistically using the IBM SPSS Statistics 26 software. The Shapiro–Wilk test was carried out to determine the normality of the data obtained. If the data were normally distributed, it would be tested for homogeneity and continued with one-way analysis of variance (ANOVA), followed by Duncan’s test at = 0.05. If the data were not normally distributed and not homogeneous, then the data were processed using the Kruskal–Wallis method, followed by the stepwise step-down method.

## 3. Results

### 3.1. Color Change of the Red Dragon Fruit Peel Extract

**Figure 1:**
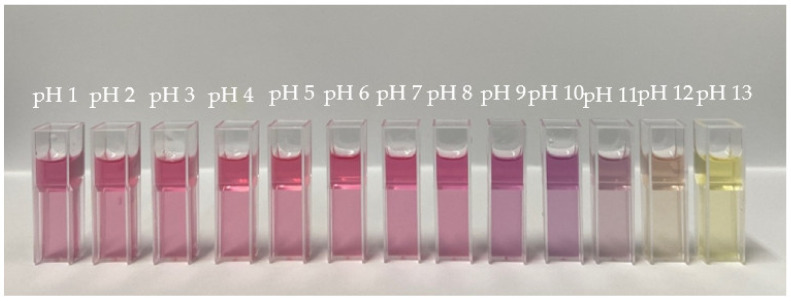
Color change of the red dragon fruit peel extract.

The total anthocyanin content of the red dragon fruit peel extract was 0.105 mg/g. The extract that was obtained was added to a pH buffer of 1–13. The results showed that the color changed from red to yellow with the increase in pH (Figure 1).

### 3.2. Composite Films

**Figure 2:**
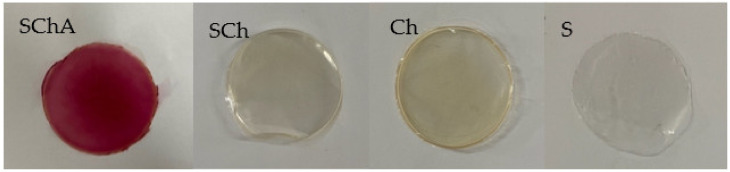
The composite films are made from: cassava starch–chitosan–anthocyanin (SChA); cassava starch-chitosan (SCh); chitosan (Ch); cassava starch (S).

The use of different formulas in the film showed the different appearances of the film. The addition of cassava starch (S) resulted in the opaque appearance of the film. Meanwhile, the addition of the chitosan (Ch and SCh) provided a more transparent, glossy, and yellowish color. Furthermore, the addition of anthocyanin extract (SchA) gave a red color (Figure 2).

### 3.3. Thickness and Mechanical Properties of Composite Films

Based on the thickness analysis, SChA film showed the highest thickness values. From the mechanical properties analysis, Ch film showed the highest tensile strength and Young’s modulus values. Meanwhile, SChA film showed the lowest tensile strength and Young’s modulus values but had the highest elongation at break value (Table 2).

### 3.4. Surface Color of Composite Films

The results of surface color analysis using a colorimeter can be seen in Table 3. SCh, Ch, and S films showed higher L* values than SChA film. SChA film showed the highest a* value, Ch film showed the highest b* value, and SChA film showed the highest ΔE value.

### 3.5. Sensitivity of Indicator Film to Ammonia Vapor

**Figure 3:**
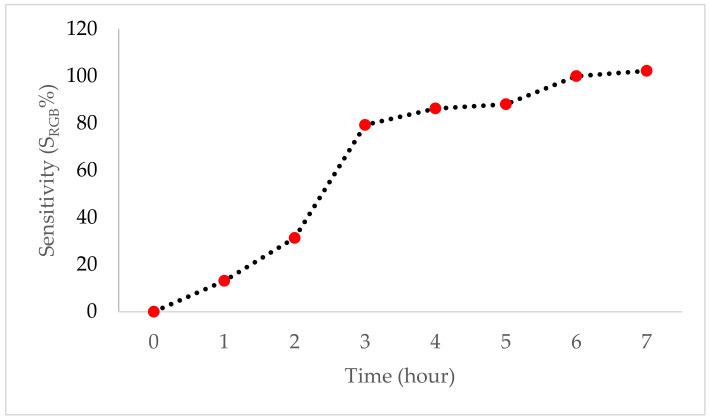
The sensitivity of SChA indicator film to 15 mM ammonia vapor.

The sensitivity of the SChA indicator film increased significantly after three hours of exposure to ammonia vapor (Figure 3). The percentage of the film sensitivity to ammonia from the first to the seventh hour was 13.06 ± 0.02%, 31.31 ± 0.09%, 79.28 ± 0.09%, 86.26 ± 0.07%, 88.06 ± 0.04%, 100 ± 0.04%, and 102.25 ± 0.04%, respectively.

### 3.6. Sensitivity of Indicator Film in Different pH Conditions

**Figure 4:**
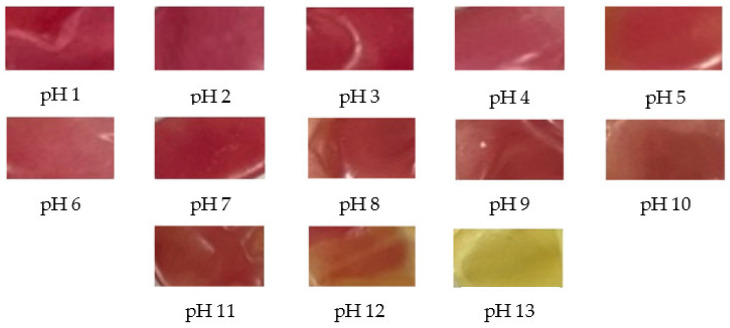
Sensitivity of SChA indicator film in different pH conditions.

The results of the sensitivity of the SChA indicator film that had been dipped in pH 1–13 buffer showed that the indicator film could change color according to the pH conditions from red to yellow (Figure 4).

### 3.7. Surface Morphology of Composite Films

**Figure 5:**
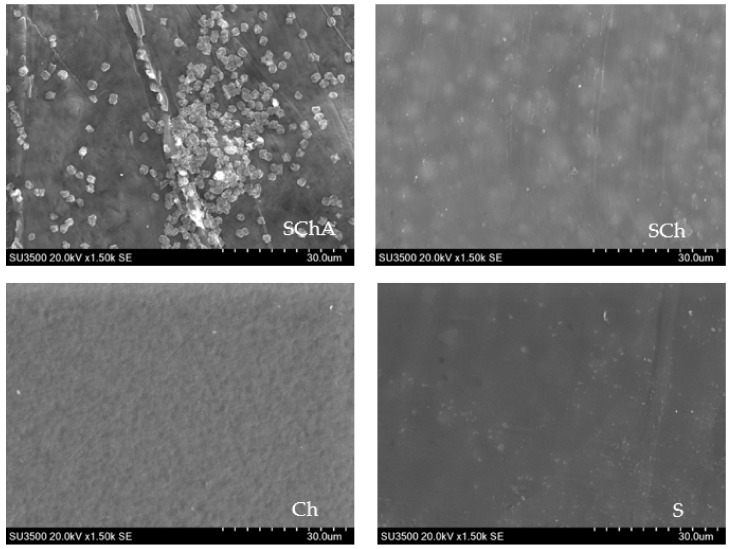
Surface morphology of composite films.

The surface morphology of the films observed using SEM showed that the SCh, Ch, and S films resulted in a flat and homogeneous surface, while the SChA indicator film showed an uneven surface (Figure 5).

### 3.8. Transparency of Composite Films

**Figure 6:**
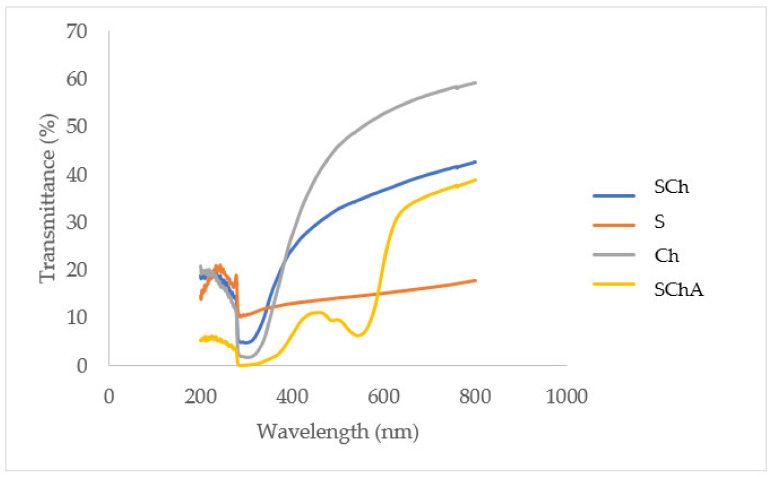
Transmittance value of composite films. The composite films are made from: cassava starch–chitosan–anthocyanin (SChA); cassava starch–chitosan (SCh); chitosan (Ch); cassava starch (S).

The transmittance values of the SChA, SCh, Ch, and S films tended to decrease at a wavelength of 300 nm and tended to increase from a wavelength of 320 nm to 800 nm. Furthermore, the SChA film showed a downward graph at a wavelength of 544 nm and then increased again from a wavelength of 545 nm (Figure 6).

The S and SChA films showed the lowest transparency values. Meanwhile, the Ch film showed the highest transparency value (Table 4).

### 3.9. Antioxidant Activity

The SChA film had the highest antioxidant activity, followed by the Ch and SCh films, respectively. Meanwhile, the S film did not show any antioxidant activity (Table 5).

### 3.10. Antimicrobial Activity

**Figure 7:**
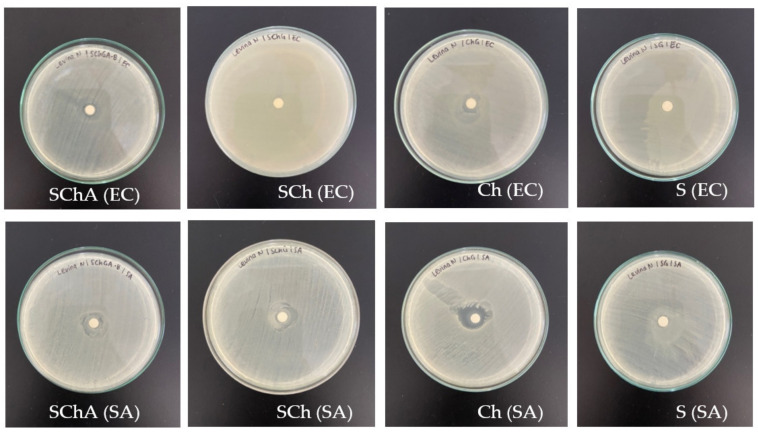
Antimicrobial activity of composite films.

The results of antimicrobial activity of the four films are indicated by the formation of a clear zone around the disc on media that had been inoculated by bacteria (Figure 7). All films containing chitosan (SChA, SCh, and Ch) showed antimicrobial activity on *Escherichia coli* (EC) as well as *Staphylococcus aureus* (SA). Meanwhile, the S film did not show any antimicrobial activity.

### 3.11. Shrimp Freshness Monitoring

**Figure 8:**
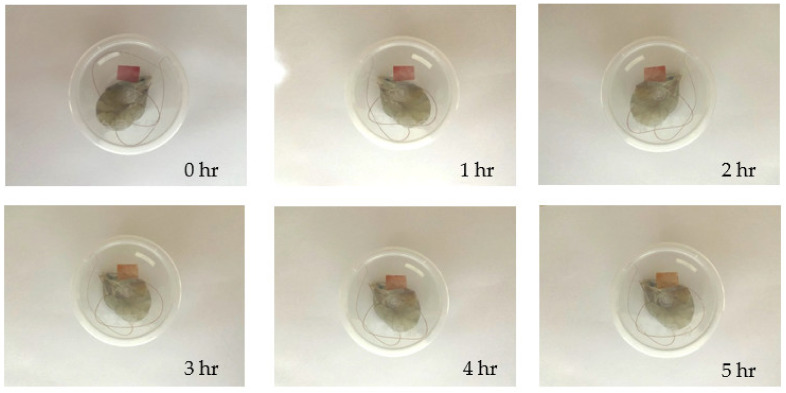
SChA indicator film on shrimp packaging at room temperature (25 °C).

The results of the application of the SChA indicator film on the freshness of shrimp at room temperature storage (25 °C) can be seen in Figure 8. The SChA indicator film shows a color change from red to pink-yellowish starting at 3 h.

**Figure 9:**
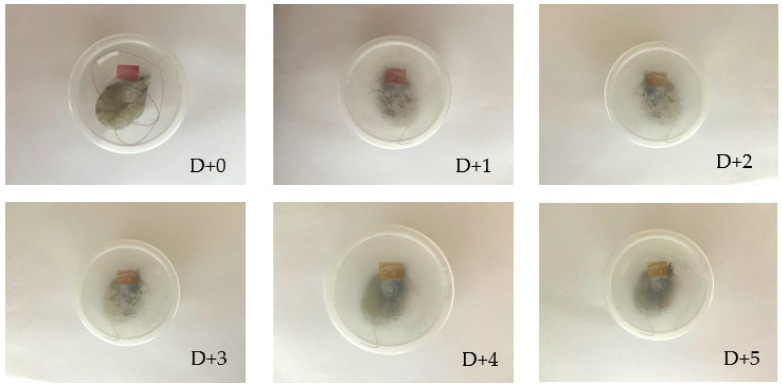
SChA indicator film on shrimp packaging at cold temperature (5 °C).

Meanwhile, the SChA indicator film showed a color change from red to pink–yellowish starting on the second day at a cold temperature (5 °C) (Figure 9).

## 4. Discussion

Based on research by Azlim et al., the total anthocyanins content in red dragon fruit peel extracted using 80% ethanol solvent was 0.32 mg/g [16]. Other research showed that the total anthocyanin content obtained using the maceration method with 96% ethanol and 1% HCl solvent followed by the distillation method to remove the solvent was 0.022 mg/g [27]. Meanwhile, the total anthocyanin content of red dragon fruit peel extract obtained in this study was 0.105 mg/g, indicating that the level obtained was in accordance with existing research.

The anthocyanin extract obtained in this study showed the ability to change color under different pH conditions. Theoretically, its ability is due to changes in its chemical structure from pink at pH 3, purple at pH 6, blue at pH 8, and yellow at pH 10 [28]. At an acidic pH, the structure of anthocyanin changes from a flavylium cation to a carbinol base. Then, at pH 6–8, the structure changes to an anhydrous quinoid base, which gives a purplish color. At alkaline pH, the structure changes to chalcone yellow, which produces a yellow color [29]. However, the color change of the extract in various pH conditions in this study not too varied. These results were influenced by the presence of another compound, namely betacyanin, in the red dragon fruit peel. Betacyanin shows a purplish red color at pH 2 to 10 and changes color to yellow at pH 12. When exposed to base conditions, betacyanin changes its structure to betalamic acid and cyclo-DOPA 5 O-glucoside, the same as if exposed to heat. According to Ardyansyah et al. [28], dragon fruit peel extract changes color from red-purple at pH 2 to 10 and becomes yellow at pH 12 when mixed with different pH buffers, where the results tend to be similar to the results in this study.

The thickness of the S film was 0.07 mm, the Ch film had a thickness of 0.08 mm, the SCh was 0.1 mm, and the indicator film was 0.12 mm [24]. The increasing film thickness was due to the SCh film consisting of three ingredients, including cassava starch solution, chitosan solution, and glycerol. Meanwhile, the SChA indicator film consists of four ingredients: cassava starch solution, chitosan solution, glycerol, and anthocyanin extract. The addition of various types of different materials makes the film thickness increase. From the measurement of mechanical properties, the highest tensile strength of the film was in the Ch film. Films made of chitosan dissolved in acetic acid had a high tensile strength when compared to chitosan dissolved in lactic acid, malic acid, and citric acid [30]. In addition, the Ch film also has a Young’s modulus or high stiffness value and a fairly low elongation at break or strain value. This shows that the Ch film is a rigid, strong, and less flexible film. This contrasts with the SChA indicator film with low-tensile-strength values, Young’s modulus, and high elongation at break values. This shows that the SChA indicator film is flexible and not rigid. The decrease in the value of tensile strength and Young’s modulus, as well as the increase in the value of elongation at break, is thought to be due to the higher water content in the film. Chitosan and starch can form intermolecular bonds between their hydrogen bonds. More specifically, the NH_2_ group in chitosan will form intermolecular bonds with the OH group in cassava starch [31]. In addition, adding anthocyanin extract will decrease the value of tensile strength and also Young’s modulus, increasing the value of elongation at break due to an increase in the interfacial force between the color extract and other polymers. This can occur due to the formation of hydrogen bonds, making the film tend to be more flexible [24].

The SChA indicator film showed the lowest L* value and the highest and positive a* value because the film formed a red color due to the presence of anthocyanin and betacyanin extracts [32]. Ch film has the highest b* value and is also positive, indicating that the color tended to be yellowish. The yellowish color formed is due to the fact that chitosan with low molecular weight has a yellowish color, as used in this study. In addition, this yellowish color will increase if glycerol is added to the films made [33,34].

The SchA indicator film was analyzed for sensitivity to ammonia to determine the ability to change color when exposed to basic nitrogen compounds, such as when shrimp freshness has decreased. The ammonia used in this analysis was 15 mM due to adjusting to the ammonia level of shrimp that were not fresh [35]. As expected, the SChA indicator film showed a color change from red to yellow from the third hour to the seventh hour. The color change that occurred is due to the volatile ammonia, which diffuses into the film matrix and combines with water molecules in the film, making it form NH_3_.H_2_O. In addition, NH_3_.H_2_O is hydrolyzed to NH_4_^+^ and hydroxyl ions (OH^−^) which are responsible for the development of alkaline conditions in the film. This process will modify the structure of anthocyanin to become chalcone, and change the betacyanin structure to betalamic acid, causing a color change [36,37]. The ability of the indicator film to change color when exposed to ammonia varies, as a study by Qin et al. showed that its indicator film made from dragon fruit peel extract and starch–polyvinyl alcohol complex could change color from red to yellow upon exposure for 20 min [37], while the SChA indicator film in this study changed color significantly to yellowish starting at the third hour of exposure. The method of film preparation could be the other factor affecting the time responsiveness of the indicator. Forghani et al. [38] reported that the indicator prepared by electrospinning showed a faster color change (10 s) than the indicator prepared by the solvent-casting method (15–40 min). The indicator film in this study can still respond to changes in pH in real time and is sensitive, meaning the indicator film can be used to monitor changes in pH.

The sensitivity of the SChA indicator film in pH 1–13 buffer showed a color change similar to the color change of the extract. The color changes that occur are not highly varied. However, it is seen that the film can change color from red to pink-yellow starting at pH 12 to 13 buffers. Moreover, the indicator film shows a pink color in pH 1 to 4 buffers and red in buffer pH values of 5 to 11. Based on research conducted by Yao et al., indicator films made from dragon fruit flesh extract tend to change color from pink at pH 3 to yellow at pH 12 when immersed in different pH buffers [39], where the results tend to be similar to the SChA indicator films at pH 12 in this research. The results showed that anthocyanin extract in the film could change color and potentially be used as indicator film [16]. The range of the color change (red to yellow in the base condition) in this research has the potential for monitoring shrimp freshness. Shrimp are one of the food products that are susceptible to spoiled, which has a pH of around 7.4 as the initial pH of fresh shrimp and will increase as its freshness deteriorates during storage [35].

The results of the morphological analysis by SEM showed that the three films (SCh, Ch, and S) had a fairly even surface when compared to the SChA film [16]. The surface of the SChA film on the SEM showed the presence of two inhomogeneous phases, indicated by conspicuous gray and white granules. In contrast to Azlim et al., the addition of color extract from dragon fruit peel caused the film to have a smooth and even surface [16]. The occurrence of these two inhomogeneous phases can occur because there are components that are not dissolved in the film solution. Based on this result, in manufacturing indicator films, it is necessary to filter the color extract before it is mixed into the film solution.

SchA, SCh, Ch, and S films tend to experience a decrease in light transmittance at a wavelength of 300 nm. However, the four films also tend to experience an increase in the transmittance percentage from 300 nm to 800 nm. This shows that the percentage of light transmittance increases in visible light. SCh, Ch, and S films tend to have high light transmittance values, indicating that the ability of the three films as a light barrier is poor. The higher the percentage of transmittance that occurs, the greater the absorption of light on the film. The SChA indicator film decreased at a wavelength of 300 nm, then rose again and fell at 545 nm. In the graph, it can also be seen that the SChA film tends to have the lowest transmittance values, especially at UV wavelengths. There are unsaturated bonds in anthocyanin and betacyanin which absorb UV radiation [10].

The transparency of the Ch film showed the highest value, which was 36.04 log T_600_/mm. This value will decrease with the addition of cassava starch solution and also anthocyanin extract. The transparency value of SCh film was 14.53 log T_600_/mm, while the SChA film shows a lower transparency value of 11.89 log T_600_/mm. The SG film has the lowest transparency value, which is 10.65 log T_600_/mm. The transparency value of Ch film tends to be high because the films made of chitosan can form a clear solution, similar to Ardyansyah et al.’s study which states that the higher the amount of chitosan added to the film, the higher the transparency will be [28]. The transparency value is also known to decrease as the film thickness increases. In addition, the S film has the lowest transparency due to the opaque character of the cassava starch solution. As is known, the opaque nature of cassava starch is due to its compact matrix. The intermolecular bonds between starch and glycerol form a more compact and opaque film [40,41].

Antioxidant activity was only found in films containing chitosan and also color extract, although the value was relatively small. This can happen because chitosan has the ability to prevent the initial oxidation step. The free NH_2_ group at the C2 position of chitosan is able to take the H^+^ group from the film solution to become NH_3_^+^ and interact with DPPH free radicals to form stable molecules [33]. The higher the molecular weight of chitosan, the higher its antioxidant activity [42]. In this study, the chitosan used was low-molecular-weight chitosan, meaning its antioxidant activity was not too high, considering that chitosan does not have hydrogen atoms that can be easily donated [43]. Anthocyanins also have high polyphenol compounds, and both pigments have the ability to donate hydrogen atoms from the hydroxyl group (OH), meaning they can bind to DPPH free radicals [44].

Antimicrobial properties were seen in all films containing chitosan (SChA, SCh, and Ch). The Ch film showed the best antimicrobial activity compared to other films, especially Gram-positive ones. The inhibition of Gram-positive bacteria by chitosan and dyes is more effective than Gram-negative bacteria because Gram-negative bacteria have a more complex outer layer consisting of several polysaccharides, proteins, and fats in addition to peptidoglycan [45]. Chitosan can better inhibit Gram-positive bacteria because it can form a polymer membrane on the cell surface, thereby inhibiting nutrients from entering the cell. Meanwhile, in Gram-negative bacteria, chitosan must enter the cell through the pervasive method [46]. Anthocyanins also have antimicrobial properties, where these dyes will damage cell walls, membranes, and the intracellular matrix of bacteria [47]. Based on this study, it can be seen that the S film does not have antimicrobial activity. This is in accordance with Valencia et al.’s study [48] which stated that cassava starch did not have antimicrobial activity and showed the highest microbial contamination. Films made of chitosan in high concentrations showed the best antimicrobial activity.

The SChA indicator film showed a color change in the third hour at room temperature storage (25 °C) and the second day at cold storage (5 °C). The color change occurs due to the presence of volatile nitrogen base components, such as ammonia, dimethylamine, and trimethylamine, which appear when the shrimp is not fresh. This will increase the pH in the environment around the indicator film, which will cause the indicator film to change color [49,50]. The nitrogen base component arises due to protein degradation in food products caused by microorganisms and enzymes. Dong et al. stated shrimp produced 16.35 mg/100 g of total volatile basic nitrogen (TVB-N) on the first day of storage at 20 °C [26]. After that, TVB-N levels increased to 208.71 mg/100 g after storage for three days. Based on these results, it can be seen that the results in this study differ from the literature because the indicator film changes color in the third hour at 25 °C storage temperature. However, the increasing temperature is known to accelerate the formation of TVB-N. Based on research conducted by Zhang et al. [25], shrimp produced 18.6 mg/100 g TVB-N on the first day of cold storage (4 °C). After 36 h of storage, TVB-N levels increased again to 31.6 mg/100 g, and at 60 h of storage, TVB-N levels increased to 56.8 mg/100 g. The results in this study were still consistent because the indicator film began to show color changes after 48 h of storage of shrimp at cold temperatures (5 °C). These results indicate that the SChA indicator film can monitor shrimp freshness effectively and in real time.

## 5. Conclusions

An indicator film based on cassava starch–chitosan incorporated with red dragon fruit peel anthocyanin was prepared using the solvent-casting method. The resulting indicator film also showed good mechanical properties, considering that the resulting film was more flexible and not rigid, meaning it can be easily applied to food packaging. In terms of functionality, the indicator film had antioxidant and antimicrobial activity, although the value was not too high. On the other hand, the film strength was relatively low, indicating the formula still needs improvement. Overall, this indicator film had good sensitivity and can change color when applied to shrimp packaging, which implies its potential as an indicator film to monitor food freshness.

## Figures and Tables

**Table 1:** Composite film formulation.

Formulation	Chitosan 1% (*w/v*) (mL)	Cassava Starch 2% (*w/v*) (mL)	Glycerol 85% (mL)	Anthocyanin Extract (mL)
SChA	7.46	7.46	0.08	5
SCh	9.96	9.96	0.08	0
Ch	19.92	0	0.08	0
S	0	19.92	0.08	0

**Table 2:** Thickness and mechanical properties of composite films.

Film	Thickness (mm)	Tensile Strength (MPa)	Elongation at Break (%)	Young’s Modulus
SChA	0.12 ± 0.00 ^d^	3.89 ± 0.15 ^a^	27.62 ± 0.57 ^b^	0.14 ± 0.01 ^a^
SCh	0.1 ± 0.01 ^c^	4.68 ± 0.31 ^b^	26.14 ± 0.92 ^b^	0.18 ± 0.00 ^b^
Ch	0.08 ± 0.00 ^b^	14.01 ± 0.44 ^d^	23.08 ± 0.76 ^a^	0.61 ± 0.00 ^d^
S	0.07 ± 0.00 ^a^	8.03 ± 0.3 ^c^	21.77 ± 1.58 ^a^	0.37 ± 0.03 ^c^

All values shown are mean ± standard deviation. Different letters in each column indicate significant differences, α = 0.05.

**Table 3:** The surface color of composite films.

Film	L*	a*	b*	ΔE
SChA	40.07 ± 0.91 ^a^	38.8 ± 1.07 ^d^	7.33 ± 1.14 ^b^	66.24 ± 1.24 ^d^
SCh	79.57 ± 0.24 ^c^	1.39 ± 0.09 ^b^	6.58 ± 0.02 ^b^	14.84 ± 0.39 ^b^
Ch	76.97 ± 0.34 ^b^	2.76 ± 0.16 ^c^	12.14 ± 0.46 ^c^	19.73 ± 0.13 ^c^
S	81.91 ± 0.26 ^d^	0.78 ± 0.04 ^a^	−1.8 ± 0.05 ^a^	12.06 ± 0.24 ^a^

All values shown are mean ± standard deviation. Different letters in each column indicate significant differences, α = 0.05.

**Table 4:** Transparency of composite films.

Film	Transparency (log T_600_/mm)
SChA	11.89 ± 0.54 ^a^
SCh	14.53 ± 0.63 ^b^
Ch	36.04 ± 1.19 ^c^
S	10.65 ± 0.17 ^a^

All values shown are mean ± standard deviation. Different letters in each column indicate significant differences, α = 0.05.

**Table 5:** Antioxidant activity of composite films.

Film	Antioxidant Activity (%)
SChA	4.90 ± 0.08 ^c^
SCh	2.52 ± 0.22 ^a^
Ch	4.46 ± 0.08 ^b^
S	-

All values shown are mean ± standard deviation. Different letters in each column indicate significant differences, α = 0.05.

## Data Availability

Not applicable.
